# Development and validation of a prognostic nomogram for early hepatocellular carcinoma treated with microwave ablation

**DOI:** 10.3389/fonc.2025.1486149

**Published:** 2025-02-28

**Authors:** Jing Zhang, Guanya Guo, Tao Li, Changcun Guo, Ying Han, Xinmin Zhou

**Affiliations:** State Key Laboratory of Holistic Integrative Management of Gastrointestinal Cancers and National Clinical Research Center for Digestive Disease & XiJing Hospital of Digestive Diseases, Air Force Medical University, Xi’an, China

**Keywords:** hepatocellular carcinoma, early stage, microwave ablation, overall survival, nomogram

## Abstract

**Objective:**

An effective model for risk stratification and prognostic assessment of early hepatocellular carcinoma (HCC) patients following microwave ablation (MWA) is lacking in clinical practice. The aim of this study is to develop and validate a prognostic model specifically for these patients.

**Methods:**

Between January 2008 and December 2018, 345 treatment-naïve patients with HCC conforming to the Milan criteria who underwent MWA were enrolled and randomly assigned to the training (n=209) and validation (n=136) cohorts. The nomogram model was constructed based on the predictors assessed by the multivariate Cox proportional hazards model and validated. Predictive accuracy and discriminative ability were further evaluated and compared with other prognostic models.

**Results:**

After a median follow-up of 59.0 months, 52.5% (187/356) of the patients had died. Prognostic factors for overall survival (OS) were α-fetoprotein (AFP), albumin-bilirubin (ALBI) score, platelets, and ablation margins, which generated the nomograms. The nomogram model consistently achieved good calibration and discriminatory ability with a concordance index of 0.64 (95% confidence interval (CI): 0.59-0.69) and 0.69 (95% CI: 0.63-0.75) in both the training and validation cohorts. The performance of the nomogram model also outperformed other prognostic models. By using the nomogram model, the patient population could be correctly divided into low- and high-risk strata presenting significantly different median OS of 105.0 (95% CI: 84.1-125.9) months, and 45.0 (95% CI: 28.0-62.0) months, respectively.

**Conclusion:**

The nomogram model based on AFP, PLT, ablation margins, and ALBI score was a simple visualization model that could stratify patients with early‐stage HCC after MWA and predict individualized long-term survival with favorable performance.

## Introduction

Although surgical resection (SR) remains the optimal treatment for patients with early-stage hepatocellular carcinoma (HCC) and preserved liver function, microwave ablation (MWA) has also been used as a first-line treatment option for early HCC as recommended by guidelines due to its minimal invasiveness, good safety and excellent reproducibility (1–4). Studies comparing the outcomes of early patients with HCC treated with SR or MWA suggested that MWA offered comparable overall survival (OS) to SR (5–7). Although MWA shows promise in treating early-stage HCC, the 5-year survival rates of patients vary greatly, ranging from 37% to 90.9% (8–10). Therefore, risk stratification and prognostic evaluation are critical for early-stage HCC.

The Barcelona Clinic Liver Cancer (BCLC) system is the most relevant and extensively validated staging system for HCC. Although the BCLC system can stratify HCC risk at different stages, it may not be sufficient to provide a personalized long-term survival prediction (11, 12). Liver functional reserve is critical in predicting the prognosis of HCC. The Child-Turcotte-Pugh (CTP) classification is the most commonly used liver function assessment. However, its predictive ability in HCC is poor and limited by the absence of cirrhosis in some patients with HCC (13). The albumin–bilirubin (ALBI) grade, a promising alternative to the CTP grade to evaluate liver function reserve, was found to be a prognostic factor for patients with HCC undergoing different treatment modalities. Platelet-albumin-bilirubin (PALBI) has shown superior accuracy compared to ALBI in predicting survival in patients with HCC, particularly in patients receiving more aggressive treatment (14). As ALBI and PALBI grades only focus on liver function parameters without tumor status, their value in personalized survival prediction is diminished (11).

The nomogram is a simple visualization tool that enables the creation of personalized prediction (15–17). Several nomogram models have been developed to predict the outcomes of patients with HCC after SR, transarterial chemoembolization (TACE), or radiofrequency ablation (RFA), showing excellent discriminative ability compared with conventional staging methods (13, 16, 18, 19). Nomogram models to predict tumor recurrence after MWA for early-stage HCC have also been reported (3, 20). Furthermore, one study reported that a nomogram model could predict the long-term survival of patients with recurrent HCC who underwent MWA (21). Two studies have reported predictive models for the long-term survival of patients with early HCC following MWA, but one included only elderly patients and the other focused on patients with HCV-associated HCC (20, 22).

In the present study, we developed and validated a nomogram model for risk stratification and personalized survival prediction for patients with early HCC after MWA.

## Materials and methods

### Patients and clinical data

We conducted a retrospective study of patients with HCC initially treated with MWA at XiJing hospital between January 2008 and December 2018. This study was approved by the ethics committee of our hospital, and patients’ written consent for inclusion was waived. The inclusion criteria were as follows: 1) solitary HCC ⩽5.0 cm in diameter, or two to three HCC tumors, each ⩽3.0 cm in diameter according to the Milan criteria; 2) no radiological evidence of extrahepatic metastasis or major vascular invasion; 3) Child-Turcotte-Pugh (CTP) class A or B liver function; 4) no previous treatment for HCC. The exclusion criteria were as follows: 1) recurrent HCC; 2) combined with other malignancies; 3) loss to follow-up within 1 month after MWA; 4) other anti-cancer treatment after MWA.

Clinical and demographic data were retrieved from medical records, such as age, sex, tumor size, tumor number, comorbidity, BCLC staging, CTP grade, albumin, total bilirubin, platelet count (PLT), international normalized ratio (INR), aspartate aminotransferase (AST), alanine aminotransferase (ALT), neutrophil count, lymphocyte count, creatinine, ALBI score (14), α-fetoprotein (AFP), AST-to-PLT ratio index (APRI), neutrophil-to-lymphocyte ratio (NLR) and PALBI score (14).

### Microwave ablation treatment

MWA was performed as previously reported (23). All MWA procedures were performed by experienced interventional radiologists. The MWA system consisted of a monopole microwave antenna and a water-cooled microwave device (ECO-100, ECO Microwave Electronic Institute; KY-2000, Kangyou Medical Instrument) (14 G). A rational MWA scheme was designed based on the findings of enhanced computed tomography (CT), magnetic resonance imaging (MRI), and contrast-enhanced ultrasonography. An ideal ablative margin was achieved to completely cover the tumor and surrounding tumor edge (≥0.5 cm), with the exception of margins situated in difficult locations—large vessels, gallbladder, or bile ducts—where individual MWA procedures were performed with the intention of minimizing the potential damage (5, 10, 24).

After the administration of local anesthesia, the MWA antenna was inserted under US guidance and introduced into the tumor to reach its deep margin. To achieve complete ablation, multiple overlapping ablation approaches were applied to the tumors. When the deep lesion site and every area of the targeted tumor were covered by hyperechoic regions on the US, the procedure was terminated. The ablation area covering the tumor and its surrounding area measured at least 0.5–1.0 cm as a “safety margin.” This measurement was determined by comparing the diameter of the hyperechoic regions after the procedure with the diameter of the tumors before treatment (25).

### Follow-up and outcomes

One month after MWA treatment, all patients underwent contrast-enhanced CT or MRI to evaluate the technical success rate and ablation margins. After the initial CT scan, patients were subsequently followed up every 3 to 6 months until death or loss of follow-up. Each follow-up visit included a physical examination, liver function tests, AFP and at least one imaging examination (abdominal contrast-enhanced CT or MRI). Patients with elevated AFP or suspicious lesions on US screening were examined by contrast-enhanced CT or MRI to confirm tumor recurrence. Nodules with equivocal imaging findings were biopsied. Patients who did not visit our hospital as scheduled were telephoned for follow-up to obtain the imaging examination, treatment information and living status. The primary endpoint of the study was OS, which was defined as the interval between the first MWA and all-cause death or the final follow-up date of 31 December 2023.

### Statistical analysis

Quantitative variables were presented as mean ± standard deviation or median with interquartile range (IQR) and compared using the Student t-test or non-parametric Mann-Whitney U test, whereas categorical variables were compared using the Chi-square test or Fisher exact test. Only variables associated with a p<0.1 identified by the univariate analysis were included in the multivariate Cox proportional hazards regression analysis to identify the independent predictors of OS after multiple imputations for missing values.

The nomogram model was then developed using a combination of backward procedure and forward stepwise elimination techniques and Akaike’s information criteria. The variance inflation factor and correlation statistics were used to evaluate collinearity between the variables. To account for nonlinearity, continuous variables were fitted using restricted cubic splines. The Hosmer−Lemeshow test and coefficient of determination (R^2^) were used to identify the goodness of fit of the nomogram model. The area under the time-dependent receiving operator characteristic curve (AUROC), concordance index (C-index) and calibration curve were used to assess the predictive accuracy and discriminative ability of the nomogram model. To reflect the clinical utility and net benefit of the model to patients, decision curve analysis (DCA) was also performed using the source file “stdca.R.” These activities utilized bootstrapping with 1000 replications. In both the training and validation cohorts, the nomogram model was compared with other prognostic models such as the BCLC staging system (11), ALBI grade (14, 26), PALBI grade (14), and NLR (27). All statistical analyses were performed using SPSS 26.0 (IBM Corp., Armonk, NY, USA) and R version 4.1.3 software (R Foundation for Statistical Computing, Vienna, Austria) with rms, pROC and ggplot2. P-values <0.05 were considered statistically significant for differences.

## Results

### Baseline characteristics

**Figure 1:**
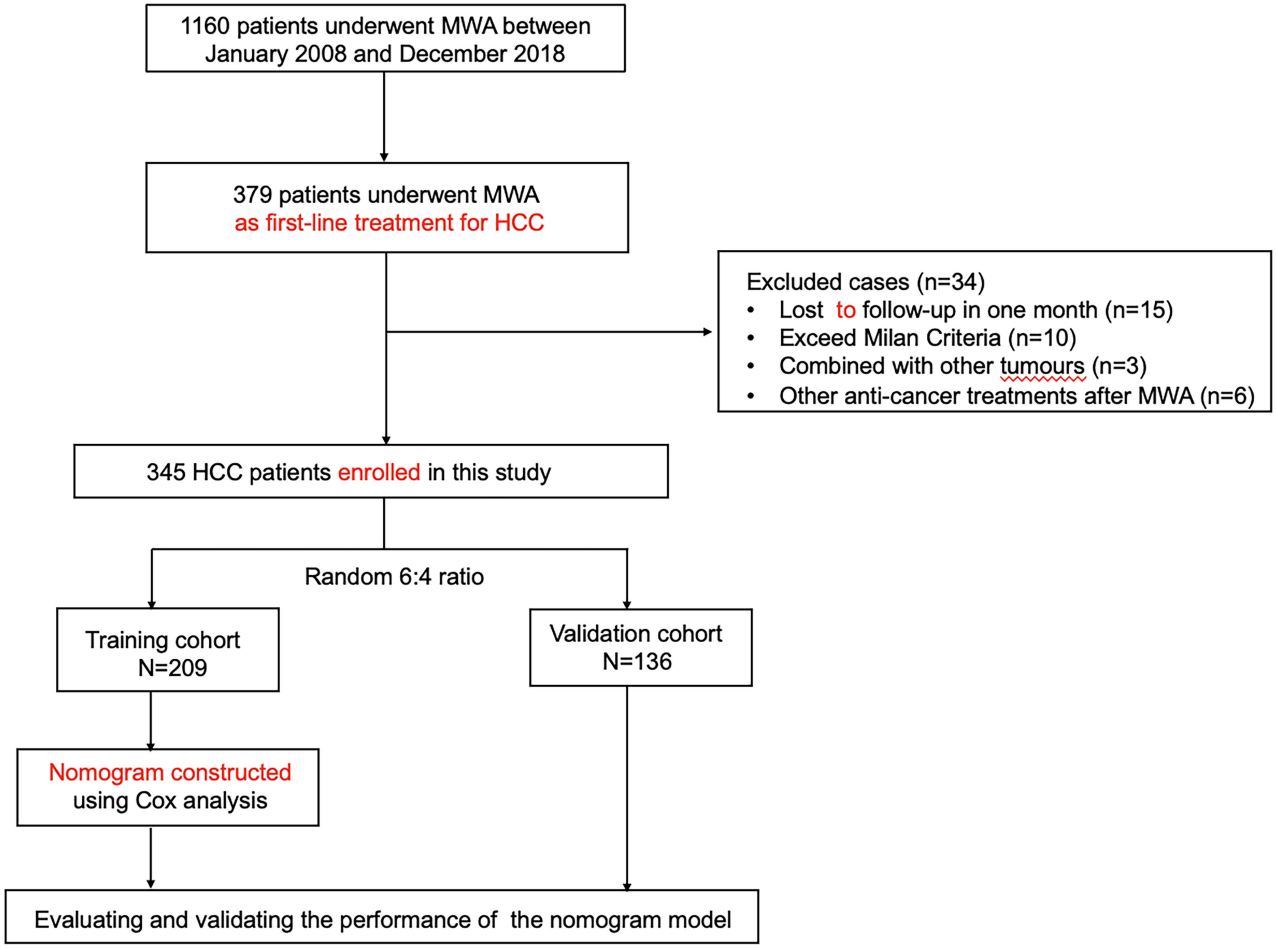
Flow chart for patient selection. HCC, hepatocellular carcinoma; MWA, microwave ablation.

A total of 345 patients with HCC were finally enrolled and randomly assigned to the training (n=209) and validation (n=136) cohorts in a 6:4 ratio using computer-generated randomization (**Figure 1**). Baseline characteristics and follow-up data were comparable between the two cohorts (all p-values>0.05) (**Table 1**). The 97.6% technical success rate in the training cohort was comparable to the 97.8% technique success rate in the validation cohort (p=0.910). After a median follow-up of 90.0 (IQR: 70.0-115.0) months, 120 (57.4%) patients in the training cohort and 69 (50.7%) patients in the validation cohort died. The OS rates had no statistically significant differences in the training and validation cohorts (p=0.263).

**Table 1:** Baseline demographics, clinical features, and outcomes of patients with early-stage HCC.

Parameter	Entire cohort (n=345)	Training cohort (n=209)	Validation cohort (n=136)	P value
Age (y)	55.0 ± 10.1	55.4 ± 10.6	54.4 ± 9.2	0.356
Male (%)	269 (78.0)	161 (77.0)	108 (79.4)	0.602
Smoking (%)	149 (43.2)	89 (42.6)	60 (44.1)	0.824
Alcohol consumption (%)	105 (30.4)	69 (33.0)	36 (26.5)	0.231
Family history of hepatitis B (%)	99 (28.7)	59 (28.2)	40 (29.4)	0.903
Etiology, n (%)				0.823
HBV	277 (80.3)	167 (79.9)	110 (80.9)	
Non-HBV	68 (19.7)	42 (20.1)	226 (19.1)	
HBV-DNA detectable, n (%)	105 (30.4)	64 (30.6)	41 (30.1)	0.925
Comorbidities, n (%)	47 (13.6)	26 (12.4)	21 (15.4)	0.521
Child-pugh grade, n (%)				0.335
A	294 (85.2)	175 (83.7)	119 (87.5)	
B	51 (14.8)	34 (16.3)	17 (12.5)	
ALBI grade, n (%)				0.502
1	165 (47.8)	104 (49.8)	61 (44.9)	
2	162 (47.0)	93 (44.5)	69 (50.7)	
3	18 (5.2)	12 (5.7)	6 (4.4)	
PALBI grade, n (%)				0.149
1	245 (53.0)	121 (57.9)	76 (55.9)	
2	148 (32.0)	56 (26.8)	47 (34.6)	
3	69 (14.9)	32 (15.3)	13 (9.6)	
Treatment session, n (%)				0.910
1	337 (97.7)	204 (97.6)	133 (97.8)	
2	8 (2.3)	5 (2.4)	3 (2.2)	
Neutrophil- to- lymphocyte ratio	1.9 (1.3, 2.8)	1.9 (1.3, 2.6)	1.8 (1.8, 3.1)	0.738
Tumor size	2.2 (1.8, 2.7)	2.2 (1.7, 2.6)	2.4 (1.8, 2.8)	0.126
Tumor number, n (%)				0.726
Solitary	302 (87.5)	184 (88.0)	118 (86.8)	
Multiple	43 (12.5)	25 (12.0%)	18 (13.2)	
Adjacent to organs or vessels, n (%)				0.694
Yes	67 (19.4)	42 (20.1)	25 (18.4)	
No	278 (80.6)	167 (79.9)	111 (81.6)	
Liver cirrhosis, n (%)	295 (85.5)	180 (86.1)	115 (84.6)	0.686
Portal hypertension, n (%)	235 (68.1)	144 (68.9)	91 (66.9)	0.699
BCLC stage, n (%)				0.435
0	101 (29.3)	64 (30.6)	37 (27.2)	
A	199 (57.7)	122 (58.4)	77 (56.6)	
B	45 (13.0)	23 (11.0)	22 (16.2)	
Performance status score				0.644
0	293 (84.9)	176 (84.2)	117 (86.0)	
1	52 (15.1)	33 (15.8)	19 (14.0)	
Ablation margins, cm				0.325
≥ 0.5	302 (87.5)	180 (86.1)	122 (89.7)	
< 0.5	43 (12.5)	29 (13.9)	14 (10.3)	
APRI score	1.0 (0.5, 2.0)	1.0 (0.5, 1.9)	1.1 (0.5, 2.0)	0.261
Alanine aminotransferase (U/L)	29.0 (21.0, 43.5)	28.0 (21.0, 43.5)	31.0 (22.0, 43.8)	0.961
Aspartate aminotransferase (U/L)	31.0 (24.0, 43.0)	29.0 (23.0, 42.0)	32.0 (25.0, 44.0)	0.114
Hemoglobin (g/L)	134.0 (120.5, 149.0)	131.0 (117.0, 150.5)	137.5 (123.0, 148.8)	0.457
Platelet count (×10^9/^L)	77.0 (48.5, 125.0)	76.0 (50.0, 126.0)	79.0 (48.0, 122.8)	0.875
White blood cells (×10^9/^L)	3.6 (2.6, 5.2)	3.5 (2.6, 5.2)	3.8 (2.7, 5.2)	0.576
Lymphocytes (×10^9^/L)	1.1 (0.7, 1.5)	1.1 (0.7, 1.5)	1.0 (0.6, 1.6)	0.312
Total Bilirubin (μmol/L)	16.7 (12.8, 24.1)	17.0 (13.4, 24.9)	16.0 (12.1, 23.0)	0.209
Albumin (g/dL)	39.4 (35.2, 43.1)	40.0 (34.5, 43.5)	39.0 (35.9, 43.0)	0.183
International normalized ratio	1.2 (1.1, 1.3)	1.2 (1.1, 1.3)	1.2 (1.1, 1.3)	0.439
Creatinine level (mg/dL)	86.0 (77.0, 97.0)	86.0 (77.0, 97.0)	87.5 (77.0, 97.0)	0.977
α-fetoprotein (ng/ml)	20.7 (4.5, 222.6)	120.2 (4.9, 195.5)	22.1 (4.3, 269.7)	0.689
Length of follow-up	90.0 (82.2, 97.8)	90.0 (80.3, 99.7)	88.0 (74.9, 101.1)	0.945
Median OS	85.0 (72.6, 97.4)	78.0 (64.1, 91.9)	91.0 (76.2, 105.8)	0.263
3-year survival rate, n (%)	278 (80.9)	170 (81.3)	108 (80.1)	
5-year survival rate, n (%)	214 (62.8)	123 (59.8)	91 (67.5)	
10-year survival rate, n (%)	161 (34.3)	93 (31.3)	68 (39.0)	

Values are presented as the median (IQR) or n (%) or mean ± standard deviation.

The P value in the table refers to the comparison between the raining and validation datasets.

HBV, hepatitis B virus; ALBI, Albumin-Bilirubin; PALBI, Plate-Albumin-Bilirubin; BCLC, Barcelona Clinic Liver Cancer; APRI, Aspartate aminotransferase-to-Platelet Ratio Index.

### Development of the prognostic model

**Figure 2:**
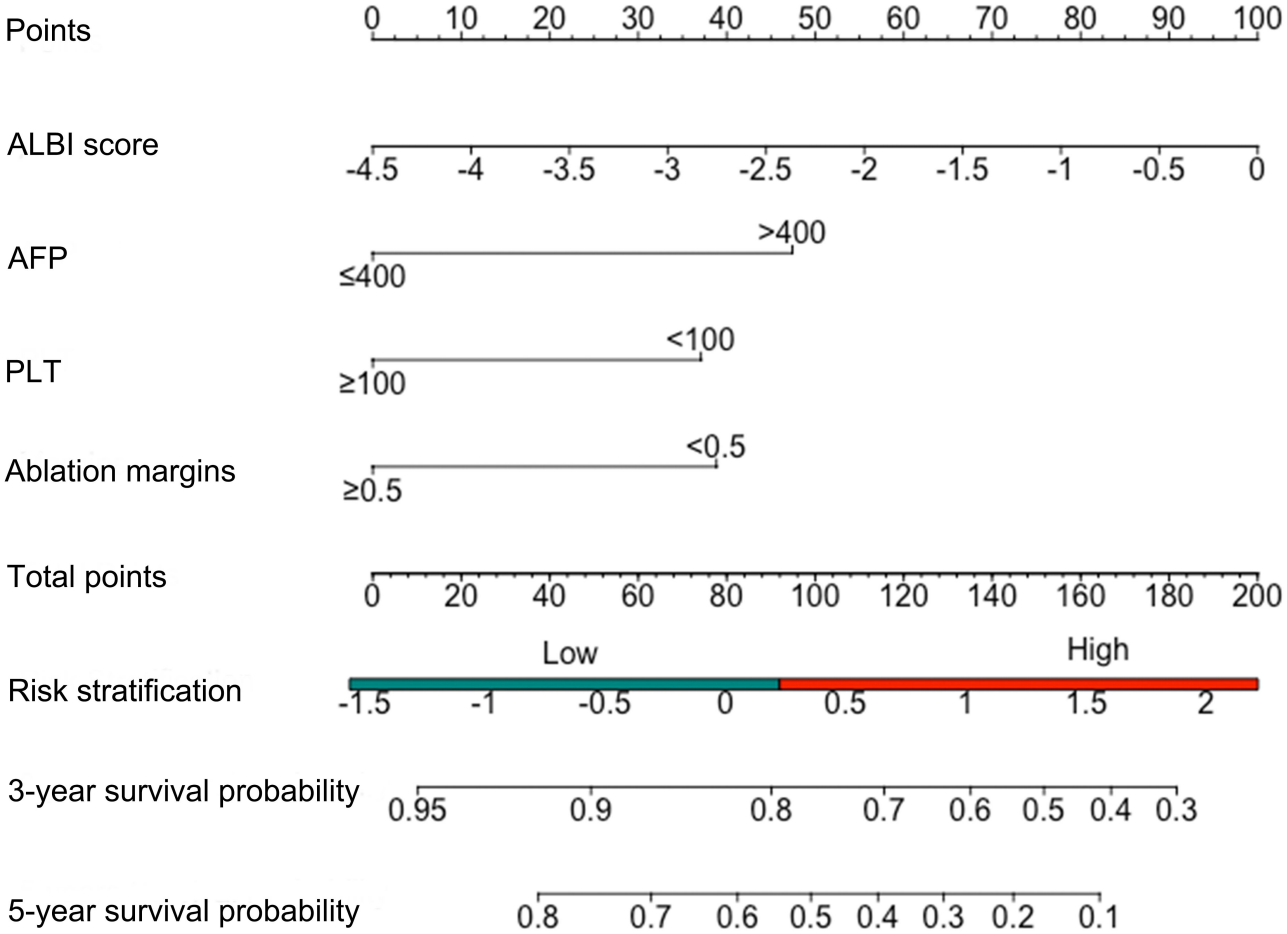
Nomogram of the new individual survival prediction model.

The multivariate Cox regression analyses after multiple imputation suggested that AFP level (hazard ratio (HR), 2.38; 95% CI: 1.41-4.01, p=0.007), ALBI score (HR, 1.50; 95% CI: 1.19-2.02, P=0.007), ablation margins (HR, 4.14; 95% CI: 1.46-11.73, P=0.007) and PLT level (HR, 1.97; 95% CI: 1.23-3.20, p=0.006) were independent prognostic factors associated with OS (**Table 2**). Restrictive cubic spline functions showed that the ALBI score presented a non-linear profile in the training and validation cohorts (non-linearity p-values: 0.016 and 0.034, respectively) (**Supplementary Figures S1A, B**). A nomogram model was developed to predict the 3- and 5-year OS rates based on the identified prognostic factors (**Figure 2**). The survival probability of individual patients at different time points after MWA could be predicted with the sum of the scores for the four factors on the point scale.

**Table 2:** Univariate and multivariate Cox analysis of risk factors associated with overall survival in the training cohort.

Characteristics	Univariate analysis		Multivariate analysis	
	HR (95% CI)	P	HR (95% CI)	P
Age (per year)	1.01 (0.99-1.02)	0.473		
Sex (male vs female)	1.25 (0.81-1.94)	0.315		
Smoking status	0.90 (0.63-1.30)	0.568		
Alcohol consumption	1.15 (0.80-1.67)	0.453		
Family history of hepatitis B	1.42 (0.98-2.07)	0.068	1.43 (0.96-2.11)	0.077
NLR	0.98 (0.87-1.11)	0.786		
APRI (>2.0 vs *≤ 2.0)*	1.41 (0.95-2.11)	0.091	1.11 (0.71-1.73)	0.640
ALBI score	1.55 (1.20-2.02)	**<0.001**	1.45 (1.08-1.95)	**0.013**
Number of tumors (multiple vs single)	0.98 (0.56-1.71)	0.937		
Tumor size (3-5 cm vs *≤ 3 cm)*	0.78 (0.43-1.42)	0.414		
Liver cirrhosis (yes vs no)	1.17 (0.68-2.00)	0.579		
Child-pugh grade (B vs A)	1.81 (1.17-2.79)	**0.007**	1.10 (0.56-2.19)	0.778
Portal hypertension (yes vs no)	1.69 (1.10-2.60)	**0.016**	1.27 (0.74-2.19)	0.384
Etiology (HBV vs. non-HBV)	0.86 (0.56-1.33)	0.493		
Adjacent to organs (yes vs no)	0.89 (0.56-1.41)	0.612		
Performance status score (1vs 0)	1.36 (0.90-2.04)	0.144		
Hemoglobin(g/L)	0.99 (0.99-1.00)	**0.032**	1.00 (0.99-1.01)	0.942
Platelet count (< 100 vs ≥100*×*10^9^/L)	1.83 (1.21-2.75)	**0.004**	2.04 (1.25-3.33)	**0.004**
White blood cells	0.90 (0.79-1.01)	0.072	1.00 (0.87-1.14)	0.955
INR	3.68 (1.71-7.92)	**0.001**	1.84 (0.59-5.75)	0.296
ALT (>40 vs ≤40U/L)	0.77 (0.51-1.16)	0.214		
AST	1.00 (0.99-1.01)	0.605		
Creatinine level	1.00 (0.99-1.01)	0.982		
TBIL (>34.1 vs *≤ 34.1μmol/L*)	1.00 (0.99-1.02)	0.444		
AFP (≥400ng/mL vs <400ng/mL)	1.65 (1.02-2.67)	**0.042**	2.34 (1.38-3.97)	**0.002**
Ablation margins (<0.5cm vs ≥0.5cm)	0.57 (0.96-2.56)	0.074	4.77 (1.65-13.79)	**0.004**

CI, Confidence interval; ALBI, Albumin-Bilirubin; NLR, Neutrophil- to- lymphocyte ratio; APRI, Aspartate aminotransferase-to-Platelet Ratio Index; HBV, Hepatitis B virus; INR, International normalized ratio; AFP, α-fetoprotein.

P values in bold indicate statistical significance.

### Performance evaluation and discriminative ability of the current model

**Figure 3:**
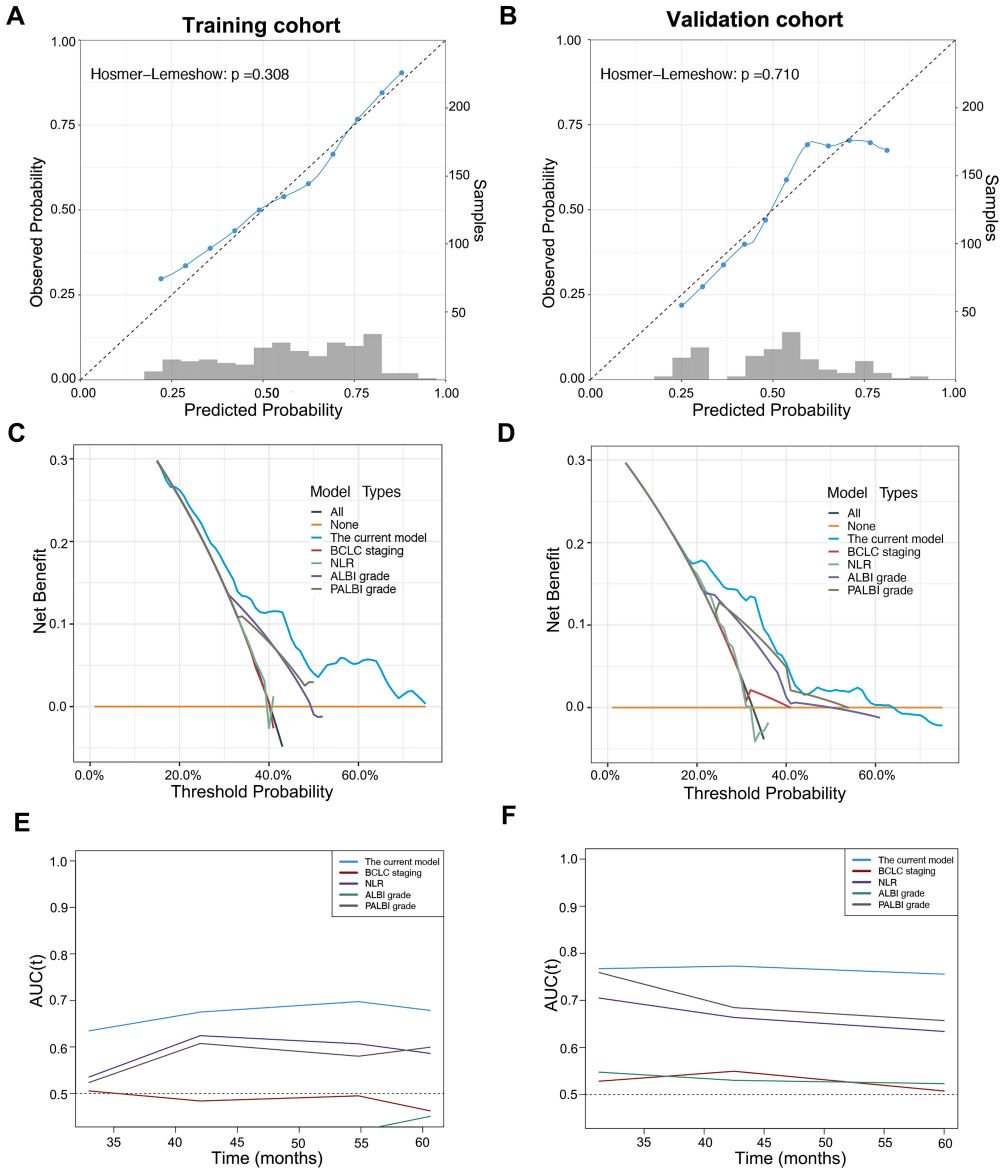
Calibration curve, decision curve analysis and time-dependent area under the receiver operating characteristic curve (AUROC) of the current model in patients with early-stage HCC after microwave ablation. Calibration curve of the current model in the training cohort **(A)** and validation **(B)** cohort. Decision curve analysis of the current model compared with other prognostic models in the training cohort **(C)** and validation cohort **(D)**. Time-dependent AUROC values of the current model compared with other prognostic models in the training cohort **(E)** and validation cohort **(F)**.

The C-index value of the current model for OS prediction was 0.64 and 0.69, respectively, in the training and validation cohorts and the calibration curves were close to the ideal diagonal line (**Figures 3A, B**). DCA demonstrated a higher net benefit of the current model than other prognostic models (**Figures 3C, D**). Additionally, the Hosmer−Lemeshow test confirmed that the current model was a good fit (p=0.308 and p=0.710 in the training and validation cohorts, respectively). The performance and discriminative ability of the current model were compared with other prognostic models, such as the ALBI grade, PALBI grade, BCLC staging system and NLR. The 3- and 5-year AUROC values and the C-index of the current model were higher than other prognostic models, which remained consistent in the validation cohort, indicating good performance and discriminative ability (**Table 3**, **Figures 3E, F**).

**Table 3:** Comparison of performance and discriminative ability between the current model and other prognostic models.

Cohorts	Models	3-yr AUROC (95% CI)	5-yr AUROC (95% CI)	C-index (95% CI)
Training cohort	The current model	0.64 (0.54~0.73)	0.69 (0.61~0.76)	0.64 (0.59~0.69)
	ALBI grade	0.59 (0.49~0.67)	0.59 (0.51~0.66)	0.58 (0.53~0.63)
	BCLC staging system	0.51 (0.42~0.60)	0.47 (0.40~0.54)	0.49 (0.44~0.54)
	NLR	0.42 (0.33~0.52)	0.44 (0.36~0.52)	0.47 (0.41~0.52)
	PALBI grade	0.55 (0.47~0.64)	0.60 (0.53~0.68)	0.57 (0.52~0.62)
Validation cohort	The current model	0.78 (0.70~0.86)	0.76 (0.67~0.84)	0.69 (0.63~0.75)
	ALBI grade	0.70 (0.60~0.79)	0.63 (0.55~0.72)	0.62 (0.56~0.69)
	BCLC staging system	0.56 (0.44~0.68)	0.55 (0.44~0.63)	0.53 (0.46~0.60)
	NLR	0.51 (0.39~0.64)	0.56 (0.46~0.67)	0.50 (0.43~0.58)
	PALBI grade	0.60 (0.50~0.70)	0.56 (0.47~0.65)	0.62 (0.56~0.69)

AUROC, Area under the receiver operating characteristic curve; CI, Confidence interval; ALBI, Albumin-Bilirubin; PALBI, Plate-Albumin-Bilirubin; NLR, Neutrophil- to- lymphocyte ratio; BCLC, Barcelona Clinic Liver Cancer.

**Figure 4:**
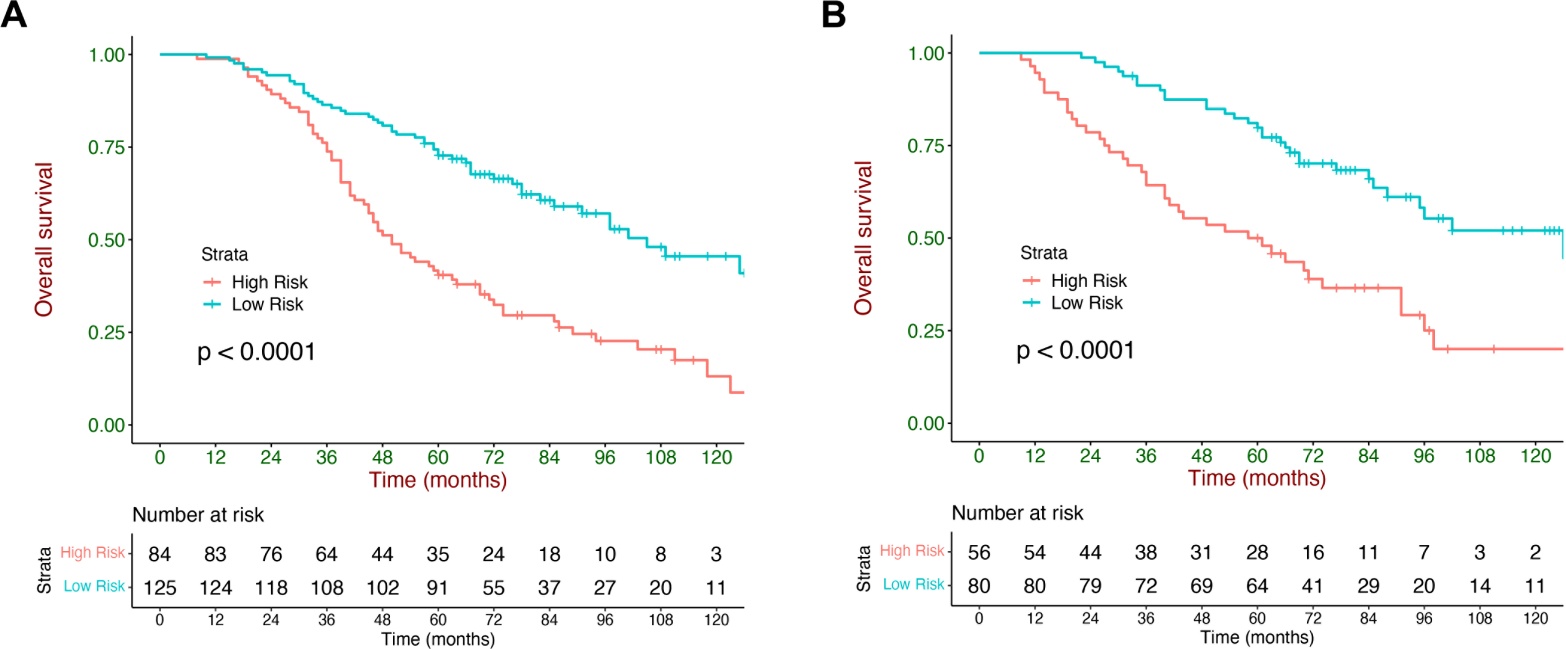
Kaplan–Meier overall survival (OS) curves stratified by the current model. Kaplan–Meier curves of OS stratified by the current model in the training cohort **(A)** and validation **(B)** cohort.

The X-tile was adopted to generate an optimal cutoff value (87), which divided the validation cohort into 2 strata with a highly different survival probability: low-risk (score <87, n=80) and high-risk (score ≥87, n=56). The median OS of the low- and high-risk strata was 126.0 (95% CI: 75.2-176.9) months and 58.0 (95% CI: 33.6-82.4) months, respectively. The survival curves were significantly different between the two strata in the training and validation cohorts (p <0.001, **Figures 4A, B**).

### Subgroup analysis

**Figure 5:**
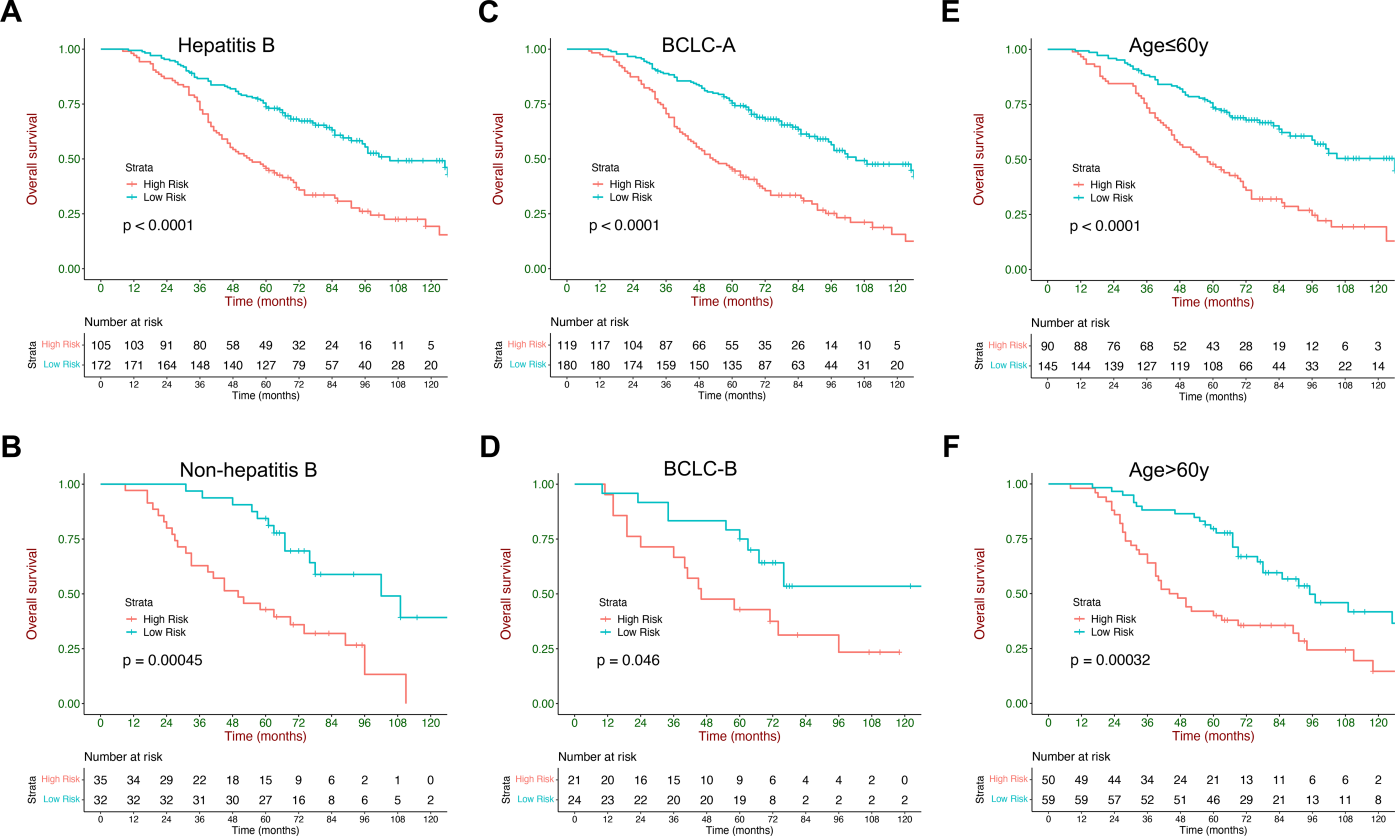
Kaplan–Meier curves of overall survival stratified by the current model in subgroups: **(A, B)** different etiologies (HBV and other etiology), **(C, D)** ages (<60 y and ≥60 y), **(E, F)** and sex (female subjects and male subjects).

The current model could also stratify patients with HCC into low- and high-risk strata across subgroups with different etiologies (HBV and other etiology), age (≤60 y and >60 y), and BCLC staging system (A and B), and exhibited consistent performance in these populations (**Figure 5**). Rates of BCLC stage A and B within each stratum are listed in **Supplementary Table S1**. Median survival and HRs with 95% CI of the low- and high-risk strata in different subgroups are listed in **Supplementary Table S2**.

## Discussion

In this retrospective observational study, we developed and validated a nomogram model including 4 items (AFP, ALBI score, ablation margins and PLT) to predict the individual outcomes in patients with early HCC after MWA. The nomogram model could accurately stratify patients into two prognostic strata with significantly distinct OS. The nomogram model showed good performance and discriminative ability and outperformed other prognostic models in predicting the long-term survival of patients with early HCC. The strength and novelty of this study lie in:1) developing the first prognostic model specifically for patients with HCC after MWA rather than unrelated to therapy; 2) The predictors are objective, routinely available and easily measured; and 3) The nomogram could be easily applied for individual survival prediction and risk stratification.

To date, some factors associated with poor outcomes in ablation have been reported. The most consistently demonstrated risk factors for poor OS included liver dysfunction, high AFP level and low PLT count. Compared with advanced tumor stages, liver function reserve is more important in patients with early HCC (13). ALBI grade incorporating both serum albumin and bilirubin could be a simple and objective method to evaluate liver function with good performance in patients with HCC. ALBI grade could predict the survival of patients with HCC across different BCLC staging and treatment modalities (14). According to previous studies, ALBI grade has been identified as a categorical predictor of long-term survival before ablation (13, 21). However, few patients with early-stage HCC had ALBI grade 3, which might weaken the statistical power of the nomogram, especially when the number of patients with ALBI grade 3 was small (13). In the present study, only 12 patients with HCC were in ALBI grade 3. Therefore, we used the ALBI score rather than the ALBI grade as a prognostic component. In recent years, the ablation margins, defined as the minimal margin distance that is measured between the area of tissue necrosis and the tumor edge, has been proposed as an independent prognostic factor (28). Recently published retrospective studies reported insufficient ablative margin as an important therapeutic predictor of mortality after ablation (28, 29), while ablative margin ≥5 mm could improve the survival outcomes (30). In the present study, we identified ALBI score, AFP, PLT, and ablation margins as the independent prognostic factors for OS.

Based on these factors, we developed a nomogram model to predict the 3- and 5-year survival in patients with early HCC after MWA. The performance and discriminative ability of the nomogram model was higher than that of other prognostic models, which was confirmed in the validation cohort. In terms of risk stratification, by forming low-risk (score <87) and high-risk (score ≥87) groups, the nomogram model could provide the survival probability prediction at different time points and divide the risk stratification with distinct OS, which was essential for the implementation of a surveillance program after MWA.

The BCLC staging system has been extensively validated and recommended for prognostic prediction and treatment allocation of HCC. However, the BCLC system is unable to stratify the survival probability of patients undergoing identical treatment. This BCLC staging system was less effective than our nomogram model in predicting OS (C-index: 0.53 vs 0.69), and its discriminative ability was less useful for patients with early HCC after MWA. Our nomogram model could differentiate BCLC A or B patients into low- and high-risk groups. The data implied that not every BCLC A or B patient was the same. The heterogeneity in early-stage HCC harbors the potential to alter HCC prognosis and surveillance strategy. For example, 40% of BCLC stage A patients and 44.5% of BCLC stage B patients were classified as high-risk strata; these patients can be considered to have similar OS rates. In addition, we also analyzed the discriminative ability of the nomogram model in patients with different ages and etiologies and found that these patients could also be divided into two strata with different OS. This result indicated that our nomogram model had a stable predictive ability of survival outcomes for patients with early-stage HCC after MWA and had great potential applications in clinical practice.

Tumor recurrence is also a critical concern that heavily influences the long-term survival of patients with HCC. We also performed multivariate analyses on factors affecting HCC recurrence. We found that only 2 factors, ablation margins and AFP level, were independent predictors of tumor recurrence (**Supplementary Table S3**). However, these two factors were not included in the nomogram model due to a lack of predictive power for survival. A possible explanation for this may be that patients with HCC underwent regular follow-up after MWA, and the majority of recurrent HCC were at an early stage, which could be treated with potentially curative methods.

For clinical application, our nomogram model could predict individualized long-term survival after MWA. This is of great importance for clinical practice. The model could identify those at high risk for poor clinical outcomes and help design an optimal surveillance strategy. A recent study evaluated the efficacy of atezolizumab plus bevacizumab in preventing recurrence of HCC in patients at high risk of recurrence after curative-intent resection or ablation and found that adjuvant therapy improved recurrence-free survival (31). With the current model, randomized controlled trials could be designed to see whether adjuvant therapy could improve overall survival in patients after MWA after stratification for long-term survival.

There are several limitations to our study. First, as a single-center retrospective study in an area where HBV is prevalent, selection bias could not be completely avoided. Although subgroup analysis by etiology suggested that our nomogram model could be effective for patients other than HBV-related HCC, its prognostic value in patients with HCC with other etiologies needs to be validated by further studies. Second, the samples from the current cohort could only be regarded as representative of the northwestern region of China, therefore a multi-center prospective study should be conducted to evaluate the prognostic accuracy. Third, our study was limited by the lack of CT and 3D software evaluation of the ablation margins, which have been demonstrated to be more accurate in assessing the successful treatment of MWA (32, 33). Finally, because this study was retrospectively designed, it is inevitable that there would be OS confounders and insufficient adherence to the follow-up protocol after MWA.

## Conclusions

In conclusion, we developed and validated a nomogram model including AFP, PLT, ablation margins and ALBI score that could perform risk stratification and predict individual survival of early HCC patients after MWA with favorable performance and discrimination. Further validation in patients with different etiologies is still needed.

## Data Availability

The data that support the findings of this study are available from the Department of Gastroenterology, XiJing Hospital. Restrictions apply to the availability of these data, which were used under license for this study. Data are available from the corresponding authors with the permission of XiJing hospital. Requests to access the datasets should be directed to zhouxmm@fmmu.edu.cn.
