# Supplementary material for: Development and validation of a prognostic nomogram for early hepatocellular carcinoma treated with microwave ablation
Source: Front Oncol. 2025 Feb 28;15:1486149. doi: 10.3389/fonc.2025.1486149 (PMC11906293; doi:10.3389/fonc.2025.1486149)
Supplement: Supplementary file 1 [file DataSheet1.docx]

**Table S1** Rates of BCLC-A and BCLC-B patients in the cohort

| **Cohort** | **BCLC-A stage**  **Number (%)** | **BCLC-B stage**  **Number (%)** |
| --- | --- | --- |
| **Entire (n=345)** | 300 (87.0) | 45 (13.0) |
| Low risk (n= 205) | 180 (87.8) | 25 (12.2) |
| High risk (n= 140) | 120 (85.7) | 20 (14.3) |
| **Training (n= 209)** | 186 (89.0) | 23 (11.0) |
| Low risk (n= 125) | 110 (88.0) | 15 (12.0) |
| High risk (n=84) | 76 (90.5) | 8 (9.5) |
| **Validation (n=136)** | 114 (83.8) | 22 (16.2) |
| Low risk (n=80) | 70 (87.5) | 10 (12.5) |
| High risk (n=56) | 44 (78.6) | 12 (21.4) |

Abbreviation: BCLC, Barcelona Clinic Liver Cancer

**Table S2** Ninety-five percent CIs for median survival and hazard ratio in subgroups

| **Cohort** | **Median survival**  **(95%CI), months** | **P-value for survival** | **HR (95% CI)** | **P-Value for HR** |
| --- | --- | --- | --- | --- |
|  |  |  |  |  |
| **HBV** |  |  |  |  |
| Low risk | 105.0 (81.6, 128.4) | P<0.001 | 0 (reference) | P<0.001 |
| High risk | 53.0 (41.5, 64.5) |  | 2.42 (1.75-3.34) |  |
| **Other aetiology** |  |  |  |  |
| Low risk | 102.0 (64.0, 140.2) | P<0.001 | 0 (reference) |  |
| High risk | 45.0 (28.8, 61.2) |  | 2.23 (1.65, 6.35) | P=0.001 |
| **Age ≤60 y** |  |  |  |  |
| Low risk | 126.0 (91.5, 160.5) | P<0.001 | 0 (reference) | P<0.001 |
| High risk | 58.0 (44.6, 71.4) |  | 2.51 (1.76-3.59) |  |
| **Age >60 y** |  |  |  |  |
| Low risk | 97.0 (70.6, 123.4) | P<0.001 | 0 (reference) | P<0.001 |
| High risk | 41.0 (31.1, 50.9) |  | 2.66 (1.63-4.37) |  |
| **BCLC-A** |  |  |  |  |
| Low risk | 105.0 (81.1, 128.9) | P<0.001 | 0 (reference) | P<0.001 |
| High risk | 52.0 (40.4, 63.6) |  | 2.53 (1.86-3.45) |  |
| **BCLC-B** |  |  |  |  |
| Low risk | 103.0 (76.0, NA) | P=0.014 | 0 (reference) | P=0.019 |
| High risk | 45.0 (59.8, 88.2) |  | 2.70 (1.18-6.21) |  |

Abbreviation: HBV, hepatitis B virus; BCLC, Barcelona Clinic Liver Cancer.
