# Supplementary material for: Development and validation of a prognostic nomogram for early hepatocellular carcinoma treated with microwave ablation
Source: Front Oncol. 2025 Feb 28;15:1486149. doi: 10.3389/fonc.2025.1486149 (PMC11906293; doi:10.3389/fonc.2025.1486149)
Supplement: Supplementary file 2 [file DataSheet2.docx]

**Supplementary Table S3** Univariate and multivariate Cox analysis of risk factors associated with recurrence-free survival in the training cohort

| characteristics | Univariate analysis | | Multivariate analysis | |
| --- | --- | --- | --- | --- |
|  | HR (95% CI) P | | HR (95% CI) P | |
| Age (per year) | 1.01 (0.99-1.02) | 0.208 |  |  |
| Sex (male vs female) | 1.27 (0.87-1.85) | 0.217 |  |  |
| NLR (>2.0 vs ≤2.0) | 1.35 (0.99-1.84) | **0.061** |  |  |
| APRI (>2.0 vs ≤2.0) | 1.05 (0.73-1.52) | 0.782 |  |  |
| ALBI score | 1.07 (1.83-1.43) | 0.620 |  |  |
| Tumour number (multiple vs single) | 0.89 (0.55-1.71) | 0.937 |  |  |
| Tumour size (3-5 cm vs ≤ 3 cm) | 1.03 (0.63-1.69) | 0.903 |  |  |
| Liver cirrhosis (yes vs no) | 0.99 (0.63-1.55) | 0.951 |  |  |
| Child-pugh grade (B vs A) | 1.05 (0.70-1.59) | 0.802 |  |  |
| Portal hypertension (yes vs no) | 1.13 (0.80-1.59) | 0.507 |  |  |
| Aetiology (HBV vs. non-HBV) | 1.32 (0.89-1.97) | 0.169 |  |  |
| Adjacent to organs (yes vs no) | 1.07 (0.83-1.39) | 0.602 |  |  |
| Performance status score (1vs 0) | 1.28 (0.91-1.80) | 0.150 |  |  |
| Hemoglobin(g/L) | 1.00 (0.99-1.01) | 0.883 |  |  |
| Platelet count (< 100 vs ≥100×10^9^/L) | 1.25 (0.89-1.74) | 0.199 |  |  |
| White blood cell | 0.98 (0.89-1.08) | 0.721 |  |  |
| INR | 1.27 (0.61-2.66) | 0.524 |  |  |
| ALT | 1.00 (0.99-1.00) | 0.384 |  |  |
| AST | 1.00 (0.99-1.01) | 0.605 |  |  |
| Creatinine level | 1.00 (0.99-1.01) | 0.709 |  |  |
| TBIL (>34.1 vs ≤ 34.1μmol/L) | 0.74 (0.43-1.25) | 0.259 |  |  |
| AFP (≥400ng/mL vs <400ng/mL) | 1.73 (1.12-2.68) | **0.014** | 1.72 (1.11-2.67) | **0.015** |
| Ablation margins (<0.5cm vs ≥0.5cm) | 2.80 (1.84-4.28) | **<0.001** | 2.80 (1.83-4.28) | **<0.001** |

Abbreviation: CI, Confidence interval; ALBI, Albumin-Bilirubin; NLR, Neutrophil to lymphocyte ratio; APRI, Aspartate aminotransferase-to-Platelet Ratio Index; HBV, Hepatitis B virus; INR, International normalized ratio; AFP, α-fetoprotein.

The bold P values represent the statistical significance.
