# Supplementary figures and images for: Development and validation of a prognostic nomogram for early hepatocellular carcinoma treated with microwave ablation
Source: Front Oncol. 2025 Feb 28;15:1486149. doi: 10.3389/fonc.2025.1486149 (PMC11906293; doi:10.3389/fonc.2025.1486149)

RCS

HR (95%CI)

The current model

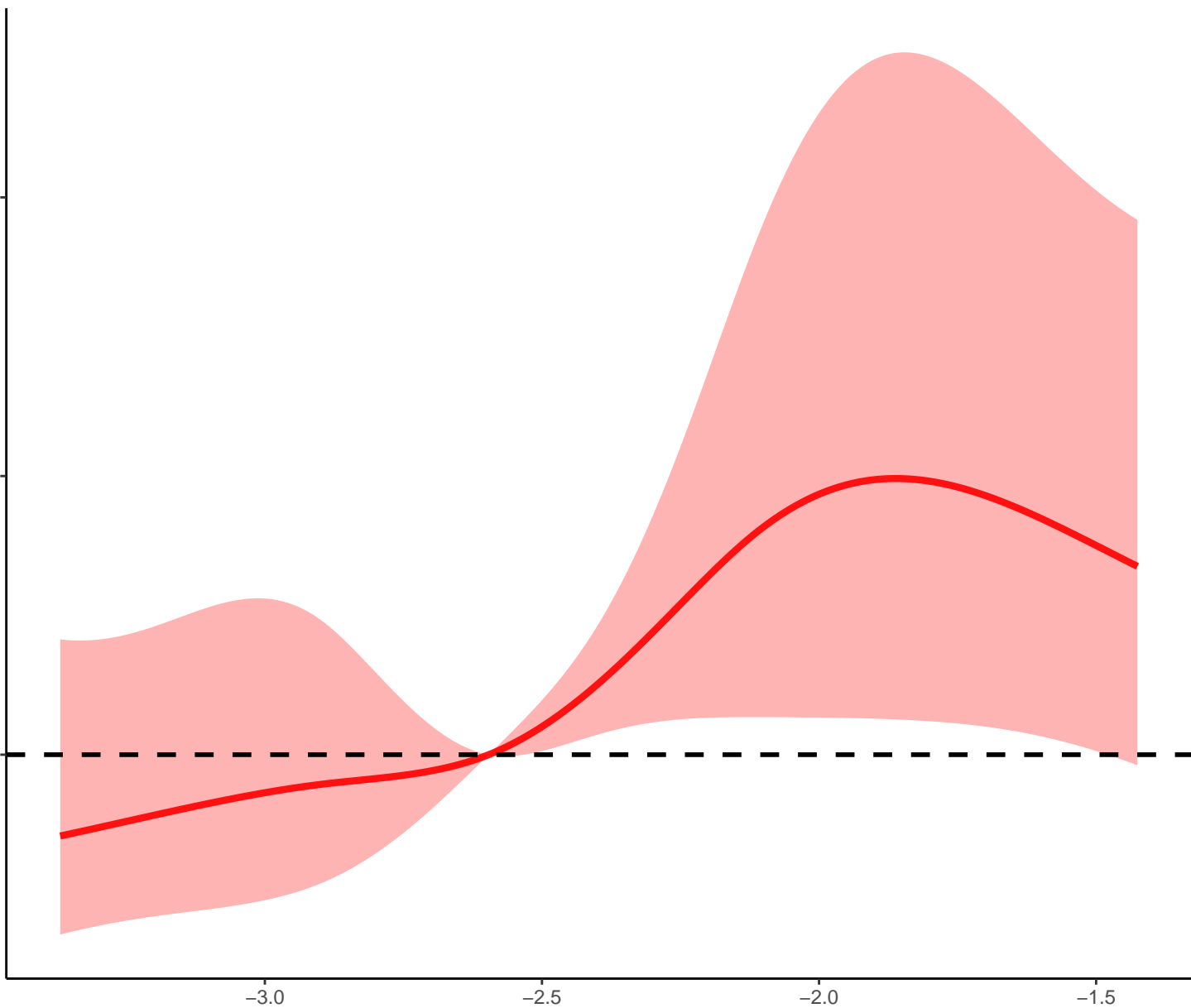

Supplement: Supplementary file 3 [file DataSheet3.pdf]
